# Safflower Seed Oil, Containing Oleic Acid and Palmitic Acid, Enhances the Stemness of Cultured Embryonic Neural Stem Cells through Notch1 and Induces Neuronal Differentiation

**DOI:** 10.3389/fnins.2017.00446

**Published:** 2017-08-03

**Authors:** Majid Ghareghani, Kazem Zibara, Hassan Azari, Hossein Hejr, Farzad Sadri, Ramin Jannesar, Ghasem Ghalamfarsa, Hamdallah Delaviz, Ebrahim Nouri, Amir Ghanbari

**Affiliations:** ^1^Cellular and Molecular Research Center, Faculty of Medicine, Yasuj University of Medical Sciences Yasuj, Iran; ^2^ER045, Laboratory of Stem Cells, DSST, Biology Department, Faculty of Sciences, Lebanese University Beirut, Lebanon; ^3^Neural Stem Cell and Regenerative Neuroscience Laboratory, Department of Anatomical Sciences, Shiraz School of Medicine & Shiraz Stem Cell Institute, Shiraz University of Medical Sciences Shiraz, Iran; ^4^Department of Biology, Payame Noor University (PNU) Tehran, Iran; ^5^Department of Pathology, Faculty of Medicine, Yasuj University of Medical Sciences Yasuj, Iran

**Keywords:** embryonic neural stem cell, safflower, linoleic acid, oleic acid, palmitic acid, stemness, Linoleic acid (PubChem CID: 5280450), Palmitic acid (PubChem CID: 985), Oleic acid (PubChem CID: 445639)

## Abstract

Embryonic neural stem cells (eNSCs) could differentiate into neurons, astrocytes and oligodendrocytes. This study was aimed to determine the effect of safflower seed oil, which contains linoleic acid (LA), oleic acid (OA), and palmitic acid (PA), on cultured eNSC proliferation and differentiation, in comparison to linoleic acid alone. Results showed that safflower seed oil, but not LA, increased significantly the viability and proliferation of eNSCs. Moreover, treatment of NSCs by safflower seed oil, but not LA, resulted in a significant increase in mRNA levels of notch1, hes1, and Ki-67, and protein levels of notch intracellular domain (NICD), in comparison to controls, indicating an enhancement of stemness. Finally, safflower seed oil, but not LA, caused an increase in the number of oligodendrocytes (MBP+), astrocytes (GFAP+) and neurons (β-III tubulin+) of which only the increase in β-III tubulin positive cells was statistically significant. In summary, OA and PA, present in safflower seed oil may prove beneficial for the enhancement of eNSCs and their neuronal differentiation.

## Introduction

It is estimated that half of the neuronal membrane is composed of fatty acids, which are the major structural components of brain cells (Zerouga et al., 1991; Bourre et al., 1992; Rapoport, 2005). In fact, polyunsaturated fatty acid (PUFA) deficiency alters the composition and structure of membranes in all cell types, including those of the nervous system (Brenner, 1981; Siguel and Maclure, 1987). Different studies have investigated the effects of fatty acids on central nervous system (CNS) using cultured neural stem cells (NSCs) which differentiate into various cells including oligodendrocytes, neurons and astrocytes (Lindvall and Kokaia, 2010). NSCs can be regulated by various endogenous and exogenous factors. Manipulating endogenous factors such as genetic networks is difficult while it is relatively easier to modulate exogenous agents such as diet. Among fatty acids, poly and/or mono-unsaturated fatty acids (PUFAs and MUFAs) have been implicated as critical nutritional factors for proper neural development and function (Gordon, 1987, 1997; Hamosh and Salem, 1998).

Three main families of unsaturated fatty acids exist: n-9 MUFA, n-3, and n-6 PUFA. MUFAs could be synthesized from saturated fatty acids whereas PUFA's are formed in plants and are not synthesized in vertebrates (Goodnight et al., 1982; Ferguson, 1984; Kelly, 1984). Linoleic acid or LA (C18:2n-6) is the major n-6 PUFA, found in plant oils (e.g., soybean and corn) and is extensively used in western diets (Barnes et al., 2008). Previous studies have demonstrated that conjugated LA, a positional and geometrical isomer of LA, stimulates the differentiation of NSCs while LA did not show such an effect (Okui et al., 2011). On the other hand, the effect of oleic acid, as a main member of n-9 MUFA family and precursor of LA, on NSCs has been rarely investigated (Yuan and Bloch, 1961; Dolecek, 1992; Lands, 2005; Chua et al., 2006; Hibbeln et al., 2006; Judge et al., 2007; Okuyama et al., 2007). Indeed, studies demonstrated the beneficial role of OA on axonal, dendrite growth and also on neuronal migration (Tabernero et al., 2001; Medina and Tabernero, 2002). A recent report investigated the effect of palmitic acid (PA) on proliferation and differentiation of embryonic NSCs (eNSCs) (Wang et al., 2014).

At the molecular level, it has been shown that basic helix-loop-helix (bHLH) transcription factors play important roles in the proliferation and differentiation of NSCs. Indeed, neuronal differentiation is promoted by activator-type bHLH factors such as Mash1 and NeuroD whereas repressor-type bHLH factors, such as notch1 and hes1 promote stemness and proliferation. The balance of activity among these factors is thought to determine the cell fate (Lee, 1997; Kageyama et al., 2000, 2005).

In this study, standard Safflower (*Carthamus tinctorius* L.) seed oil was chosen as a rich source of LA. We report, for the first time, the effect of safflower seed oil on NSC proliferation and differentiation and compare this natural source of LA to a pure synthetic one.

## Materials and methods

### Oil components

The safflower seed oil species chosen was Carthamus. Tinctorius (genotype: C4110), identical to the one previously used by Sabzalian (Sabzalian et al., 2008). Chemically, this seed oil contains 73.64% linoleic acid, 15.14 % oleic acid, 5.7% palmitic acid, and a total of 2.15% for myristic (C14:0), palmitoleic (C16:1), stearic (C18:0), arachidic (C20:0), and behenic (C22:0) acids. NSCs were treated with various concentrations of LA (25 vs. 100 μM), and low or high concentrations of safflower seed oil. Low Oil concentration contained LA 25, OA 3.8, and PA 1.6 μM while high oil concentration contained LA 100, OA 15.1, and PA 6.2 μM.

### Animals

The current study was done under approved conditions by the Institutional Animal Care and Use Committee (IACUC) and Ethics Committee of Yasuj University of Medical Science which conforms to the provisions of the Declaration of Helsinki (as revised in Brazil in 2013). All efforts were made to minimize the pain and suffering of mice during all the procedures. A total of 5 mice (*n* = 5) were used in this study.

### Culture of embryonic NSCs

Primary cultures of embryonic NSCs were performed as described previously (Azari et al., 2011). Briefly, the cerebral cortices from E14 mice were micro-dissected under sterile conditions then mechanically disrupted into single cells by repeated pipetting in the serum-free neurosphere N2 medium. This medium consists of DMEM/F12 (1:1), 0.6% (w/v) glucose, 0.1125% (w/v) sodium bicarbonate, 2 mM L-glutamine, 5 mM HEPES, 100 μg/mL human apotransferrin, 20 nM progesterone, 30 nM sodium selenite, 60 μM putrescine, and 25 μg/mL insulin. Cells were then plated in T25 flasks in suspension at a density of 1 × 10^5^ cells/mL in proliferation medium consisting of the above N2 medium supplemented with 20 ng/mL basic fibroblast growth factor (bFGF; R&D Systems, USA) and 2 mg/mL heparin (Sigma-Aldrich, USA). Cells were maintained in an incubator with a humidified atmosphere containing 5% CO_2_ at 37°C for 5–6 days (Azari et al., 2011). Neurospheres were then harvested by centrifugation, dissociated using trypsin and EDTA (Sigma-Aldrich), and reseeded for the following experiments.

### Cell viability assay

Cell viability of NSCs was assessed by employing the 3-(4, 5-dimethylthiazol-2-yl)-2, 5-diphenyltetrazolium bromide (MTT) assay. Briefly, cells from primary cultures were seeded at a density of 5,000 cells onto 96-well plates and cultured in a humidified atmosphere of 5% CO_2_ at 37°C. Cells derived from neurospheres were dissociated and then seeded at a density of 5,000 cells in 96-well plates and treated for 48 h with various concentrations of LA (25 vs. 100 μM), or safflower seed oil (25 vs. 100 μM) containing also OA (3.8 vs. 15.1 μM) and PA (1.6 vs. 6.2 μM), respectively. LA and oil-containing medium were then removed, 48 h after the treatment, and wells were then gently washed twice with PBS and then 200 μl of 0.5 mg/ml MTT in PBS was added to each well. The plate was incubated at 37°C for 4 h. Then, the cells were disrupted in a solubilizing solution (1:1 ratio of dimethyl sulfoxide, DMSO, and ethanol, EtOH). The formazan dye produced by viable cells was quantified in an ELISA microplate reader at an absorbance of 460 nm. Results were expressed as OD. A total of five independent experiments were conducted.

### Neurosphere formation assay

Neurosphere-forming cells obtained from passage-1 flasks were then harvested by centrifugation, dissociated using 0.05% trypsin-EDTA (Sigma-Aldrich), and reseeded for the following experiments after determining the cell density using trypan blue exclusion assay (Azari et al., 2011). Cells were then cultured at 25 cells/μl in 0.2 ml of media in uncoated well plates and treated by different concentrations of fatty acids (each treatment concentration repeated in 15 wells). The total number of neurospheres, with a diameter of >50 μm, was counted after 6 days using an Olympus inverted light microscope, and expressed as the neurosphere-forming frequency per well.

### Real-time PCR

The NSCs were cultured and allowed to proliferate for 5 days in the above proliferation medium in the presence of different concentrations of fatty acids, as described at cell viability assay method. Total RNA from NSCs was isolated using QIAGEN RNeasy Kit (Qiagen, Japan), and then cDNA was synthesized with the High-Capacity cDNA Reverse Transcription kit (Applied Biosystems, USA). Quantitative real-time PCR (qPCR) was performed by the StepOne Real-Time PCR system (Applied Biosystems). Real-time PCR was carried out with RealQ Plus 2x Master Mix Green (Ampliqon, Denmark), according to manufacturer's instructions. The primer sequences used were the following: hes1, F: TTCCTCCCATTGGCTGAAAG and R: CCAGCTCCAGATCCAGTGTGAT; notch1, F: TGGTTCAGGGCGGTGCTCA and R: CAGACACCTGCTTCCCAAAAGG; Ki-67, F: GAGCAGTTACAGGGAACCGAAG and R: CCTACTTTGGGTGAAGAGGCTG; β-actin, F: AGATGTGGATCAGCAAGCAG and R: GCGCAAGTTAGGTTTTGTCA. The specificity of PCR products was confirmed by melting curve analysis (data not shown). The PCR conditions were as follows: initial activation at 95°C for 15 min, then 35 cycles of amplification cycles of denaturation at 95°C for 15 s, annealing at 57°C for 30 s, and extension at 72°C for 30 s. The relative changes in gene expression levels were determined by the Comparative CT (ΔΔCT) method. All reactions were performed in triplicate using β-actin as an internal control.

### Western blotting

NSCs were cultured and allowed to proliferate for 5 days in proliferation medium in the presence of different doses of fatty acids, as described in the section of cell viability assay. Cells were then homogenized on ice and lysed in a lysis buffer containing 50 mM Tris–HCl (pH 7.5), 150 mM NaCl, 0.5% deoxycholic acid, 1% Nonidet P40, 0.1% SDS, 1 mM PMSF, and 100 mg/ml leupeptin. Protein content was measured using a Bio-Rad colorimetric protein assay kit (Bio-Rad, Hercules, CA, USA). An equal amount of total protein (40 μg) was resolved on 8–15% SDS-PAGE and then transferred onto a nitrocellulose membrane. The membranes were blocked for 1 h in 5% skim milk solution, and then incubated with primary antibody against NICD (1:500, Abcam, USA) or β-actin (1:1,000, Santa Cruz Biotechnology Inc. CA, USA) for an overnight on shaker at 4°C. After washing, horseradish peroxidase-conjugated secondary antibodies were incubated at room temperature. Immunoreactive proteins were detected with an enhanced chemiluminescence western blotting detection system. Relative densities of protein bands were scanned by densitometry using MyImage (SLB, Seoul, Korea), and quantified by image analysis software for gel documentation (LabWorks Software Version 3.0, UVP Inc., CA, USA).

### NSCs differentiation

After the second passage (P2), neurospheres were mechanically dissociated and cells seeded onto poly-L-ornithine (15 mg/mL, Sigma-Aldrich) coated 12-well plates at a density of 1 × 10^6^ cells/well in N2B27 medium without bFGF and EGF. The wells were then treated with different concentrations of LA and safflower seed oil, dissolved in N2 medium containing 5% fetal bovine serum (FBS; Gibco, USA). The culture medium was changed every other day.

### Immunofluorescence (IF) analysis

After 5 days, differentiation plates were used for Immunofluorescence (IF) analysis. Cells were fixed in 12 well chamber slides using 2% paraformaldehyde for 20 min, washed with PBS, and permeabilized using 20% tween for 20 min, and then blocked in PBS, 2% triton, 5% horse serum (PBSTS) for 20 min. The cells were then incubated overnight with primary antibodies against GFAP (1:800 dilution), MBP (1:500) and β-III Tubulin (1:1,000) (all from Sigma Aldrich, USA). Slides were then washed in PBS and reacted with a secondary antibody conjugated to Alexa flour 488 and 568 (1:500 dilution) in PBSTS for 1 h for MBP/β-III Tubulin and GFAP, respectively. Cells were then imaged on an Olympus BX60 microscope with a digital camera (Spot camera, Diagnostic Instruments Inc.). Representative pictures of each well (10–12 fields/well) were taken using a fluorescent microscope (Olympus IX-71) equipped with a Canon EOS digital camera. Cell counts were performed and data presented as mean of positive cells in 10–12 fields/well.

### Statistical analysis

Results are presented as an average with the standard error of the mean (Mean ± SEM). The Kolmogorov-Smirnov test, with the Dallal-Wilkinson-Lilliefor corrected p value was used for normality assessment. A one-way analysis of variance (ANOVA) following Tukey post-test was performed for comparisons between multiple groups using Graph Pad Prism 6 software. Statistical significance is indicated by ^*^p < 0.05; ^**^p < 0.01; ^***^p < 0.001, and ^****^, *p* < 0.0001.

## Results

### Effect of LA and safflower seed oil on cell viability and proliferation of embryonic NSCs

**Figure 1:**
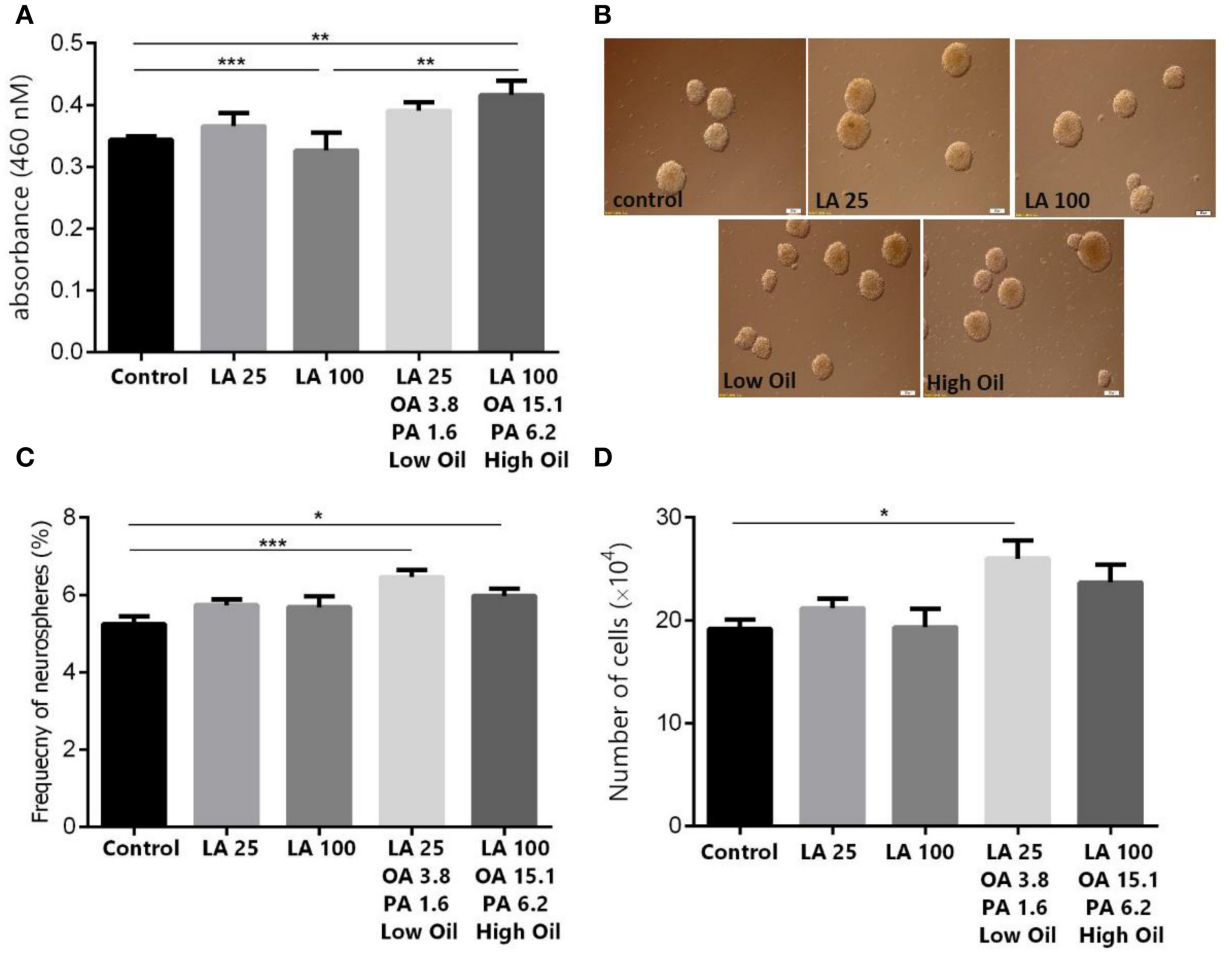
Effect of safflower seed oil on cell viability and neurosphere formation of NSCs. **(A)** Cell proliferation quantified by measuring the change in the abundance of viable cells. Neurospheres from eNSC were incubated in LA or safflower seed oil for 48 h. MTT was added to each well at 4 h before the end of the incubation. The absorbance was measured at 460 nm. Results are representatives of 5 separate experiments and reported as mean ± SEM. **(B)** Representative captures of neurospheres in different groups. Scale bars: 100 μm. **(C)** Single eNSCs at a density of 500 cells/well were cultured in normal growth medium containing DMEM/F12 supplemented with FGF for 6 days to form neurospheres. The frequency of neurosphere formation was calculated by counting neurospheres with >50 μM diameter. **(D)** Cell counts obtained from neurospheres. Data are expressed as Mean ± SEM of 15 independent experiments. A one-way analysis of variance (ANOVA) following Tukey post-test was performed to compare the mean values. ^*^p < 0.05; ^**^p < 0.01; and ^***^p < 0.001 were considered significant.

Results showed, using MTT assay, that low doses (25 μm) of LA or safflower seed oil had no significant effect on cell viability in comparison to the control (Figure 1A). In contrast, high doses (100 μm) of safflower seed oil, but not LA, demonstrated a significant increase in viable NSCs (*p* < 0.01), in comparison to the control. On the other hand, neurosphere formation reflects the self-renewal capacity of NSCs when they are plated at a very low density. In this study, NSCs formed neurospheres of various sizes with diameters ranging between 50 and >100 μm (Figure 1B). In addition, the frequency of neurosphere formation was not significantly increased (*p* > 0.05, *n* = 15) after treatment with low or high doses of LA, in comparison to the control (Figure 1C). On the other hand, low or high concentrations of safflower seed oil treatment caused a significant increase (*p* < 0.001, *p* < 0.05, respectively) in the neurosphere frequency, in comparison to the control (*n* = 15), with a maximum stimulatory effect at low concentrations. Therefore, the actual cell number resulting from the neurospheres were then calculated. Results showed that low concentrations of oil significantly increased the cell number, in comparison to the control (*p* < 0.05, Figure 1D), while this increase was not significant for high oil concentrations. It's worth noting that despite the slight increase in neurosphere number in LA treated groups, the cell count did not show any significant changes, in comparison to the control.

### Notch signaling

**Figure 2:**
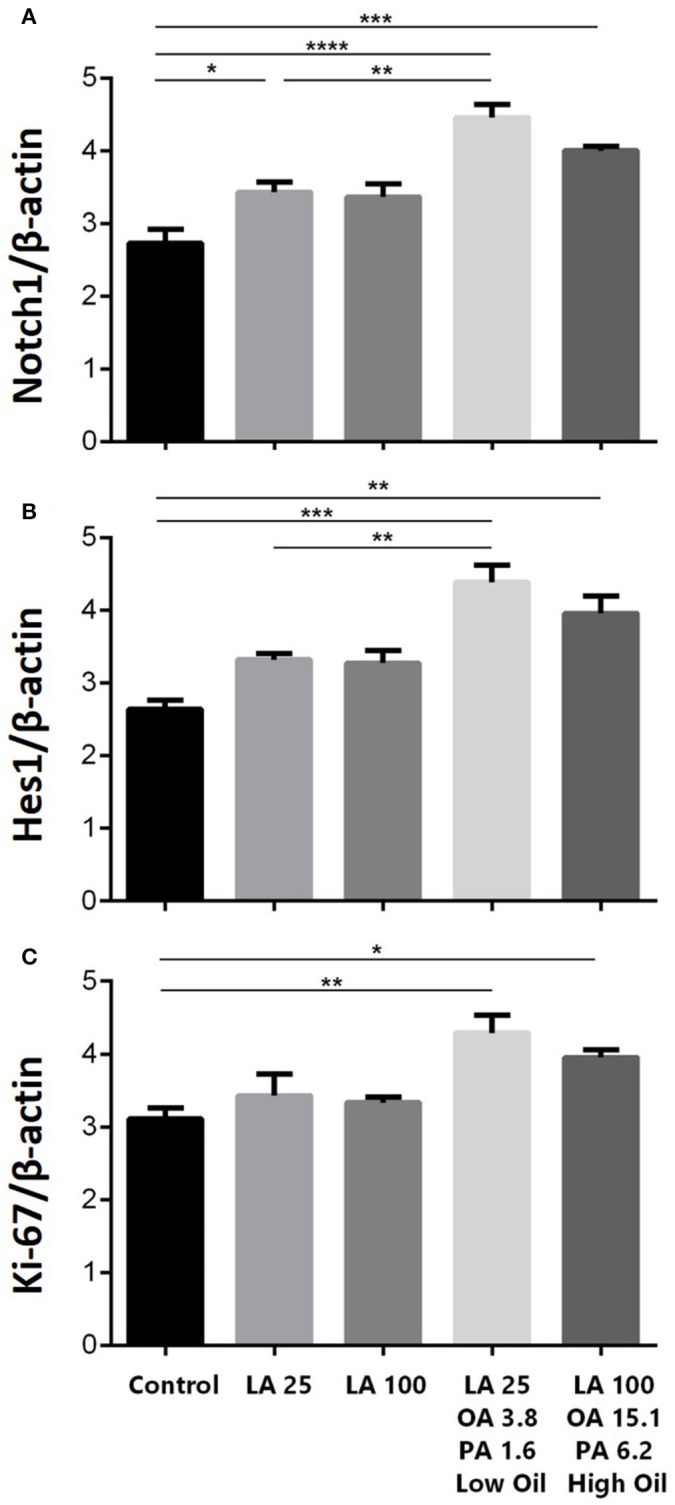
**(A)** Is for the first graph of Notch1. **(B)** is for the second graph of Hes1. **(C)** is for the third graph of Ki-67. Effect of safflower seed oil on mRNA expression levels of notch1, hes1, and Ki-67. ENSCs were cultured with low or high doses of LA or safflower seed oil for 5 days. Total RNA was prepared from each culture, cDNA synthesized and subjected to real-time PCR, using specific primers for notch1, hes1, or Ki-67. β-actin was used as an internal control. The values are expressed as the mean ± SEM. A one-way analysis of variance (ANOVA) following Tukey post-test was performed to compare the mean values. ^*^p < 0.05; ^**^p < 0.01; ^***^p < 0.001; and ^****^p < 0.0001 were considered significant.

**Figure 3:**
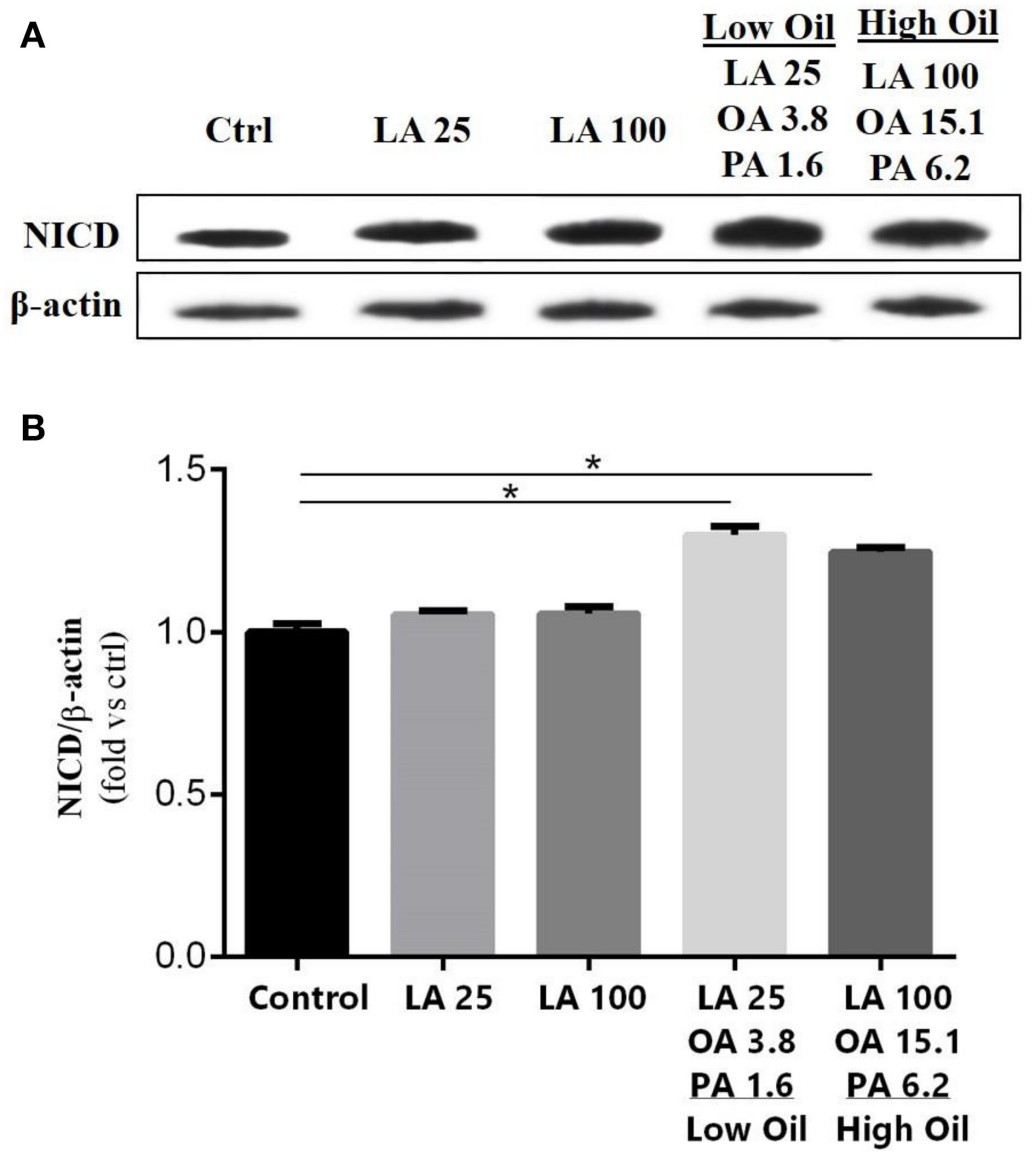
Effect of fatty acids on NICD protein expression levels. **(A)** Representative western blot showing NICD expression. **(B)** Quantification of NICD expression in all groups. β-actin was used as an internal control for normalization. Values are expressed as the Mean ± SEM. Each group included 3 replicates (*n* = 3). Statistical analyses were performed by one-way analysis of variance followed by Tukey's test. Significance is indicated by ^*^p < 0.05.

The expression of key components of proliferation markers in the Notch signaling pathway was quantified in NSCs treated with LA or safflower seed oil (Figure 2). Treatment of NSCs with low or high concentrations of safflower seed oil resulted in a significant increase (*p* < 0.0001 vs. *p* < 0.001, respectively) in mRNA expression levels of notch1, in comparison to the control (Figure 2A). On the other hand, only low concentrations of LA significantly increased notch1 mRNA levels (*p* < 0.01). Similarly, mRNA expression levels of hes1 (Figure 2B) were significantly enhanced by low or high concentrations of safflower seed oil, but not LA, in comparison to the control (*p* < 0.001 vs. *p* < 0.01, respectively). Finally, assessment of proliferation marker Ki-67 demonstrated similar results to notch1 and hes1 (Figure 2C). Indeed, low or high concentrations of safflower seed oil treatment caused a significant increase in Ki-67 mRNA expression levels (*p* < 0.01 vs. *p* < 0.05; respectively), in comparison to the control. However, LA did not have any significant effect on Ki-67 mRNA levels. It's important to note that the increase in mRNA levels was also significant when low, but also high, doses of safflower seed oil were compared to similar doses of LA (Figures 2A–C). Expression of hes1 is regulated by Notch protein, which in turn is cleaved by γ-secretase. The latter releases Notch intracellular domain (NICD) that moves into the nucleus and induces hes1 expression that inhibits the differentiation of NSCs (Gratton et al., 2003). Using western blotting, treatment of NSCs with low or high concentrations of safflower seed oil, but not LA, resulted in a significant increase (^*^, *p* < 0.05) in NICD protein expression levels, in comparison to the control (Figures 3A,B). These data are in accordance with transcriptional expression results of notch1 and hes1.

### Effect of LA and safflower seed oil on neuronal differentiation

**Figure 4:**
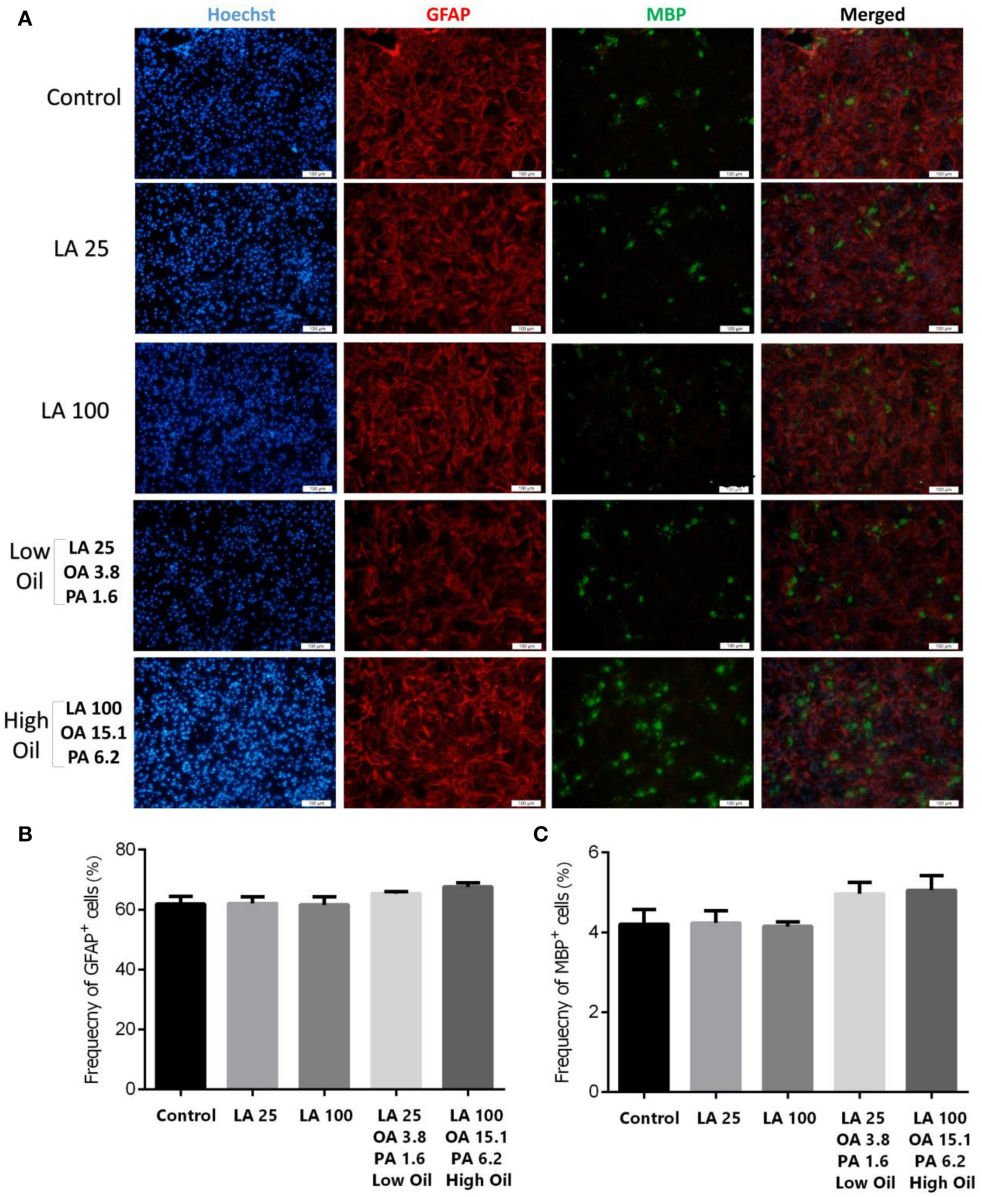
Embryonic NSC differentiation to glial and oligodendrocyte cells. **(A)** Representative images for the differentiation capacity of eNSCs toward astrocytes and oligodendrocytes. Cells were dissociated and cultured on poly-L-ornithine coated cover glasses. Cells were treated with low or high doses of LA or safflower seed oil for 6 days followed by the examination of GFAP (Red), MBP (Green), and Hoechst (nuclei, Blue), using double immunofluorescence. Scale bar is 100 μm. **(B,C)** Despite the infectiveness of LA on differentiation, low and high doses of safflower seed oil increased the frequency of GFAP+ and MBP+ cells, however; this increase was not statistically significant. Values are presented as the mean ± SEM. A one-way analysis of variance (ANOVA) following Tukey post-test was used.

**Figure 5:**
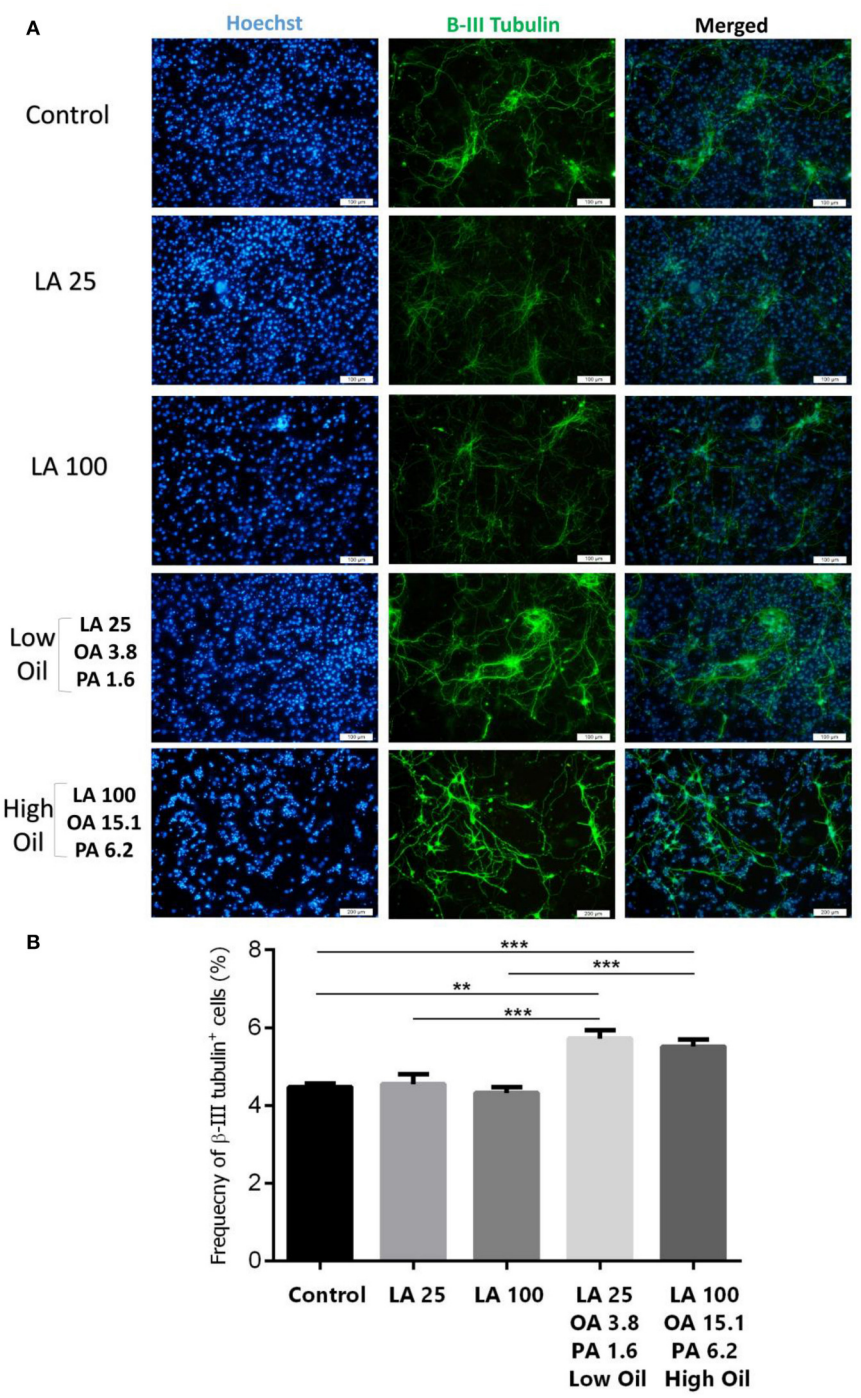
Embryonic NSC differentiation to neurons. **(A)** Representative images for the differentiation capacity of eNSCs toward neurons. **(B)** LA treatment had no effect on the differentiation of eNSCs to neurons, measured by the expression of β-III Tubulin positive cells, in comparison to controls. However, low or high doses of safflower seed oil significantly increased the frequency of β-III Tubulin cells. Values represent the frequency of positive cells in 10–12 field/well and are expressed as mean ± SEM. A one-way analysis of variance (ANOVA) following Tukey post-test was used. ^**^p < 0.01; and ^***^p < 0.001 were considered significant.

Following differentiation and treatment of cells with LA or safflower seed oil for 6 days, fluorescent images of differentiated cells were captured (Figure 4A). NSCs treated with either low (25 μM) or high (100 μM) concentrations of LA or safflower seed oil showed that the frequency of astrocytes (GFAP positive cells) was not significantly different from that of the control (Figure 4B). On the other hand, low or high concentrations of LA had no effect on differentiation of NSCs toward oligodendrocytes (MBP positive cells) whereas low or high concentrations of safflower seed oil showed a slight increase in MBP+ cells, which was not significant (Figure 4C). Finally, by quantifying the frequency of β-III tubulin positive cells, low or high concentrations of LA did not show any effect on neuronal differentiation, in comparison to the control (Figures 5A,B). However, low or high concentrations of safflower seed oil caused a significant increase in the frequency of β-III tubulin positive cells, in comparison to the control (*p* < 0.01 vs. *p* < 0.001). It's important to note that the increase in β-III tubulin positive cells was also significant (*p* < 0.001) when either concentration of safflower seed oil was compared to its similar dose of LA (Figure 5B).

Taken together, these observations suggest that the use of low or high concentrations of safflower seed oil plays a major role in the differentiation of NSCs toward neuronal cells.

## Discussion

In this study, we have chosen safflower seed oil because of its high levels of both linoleic acid (LA, 73.64%) and oleic acid (OA, 15.14%) and its low proportion of palmitic acid (PA, 5.7%) with ratios of 12.91, 2.65, and 1; respectively. We demonstrated that an increase in cell viability was dependent on the dose of safflower seed oil, which also caused a significantly higher growth of eNSCs, in comparison to pure synthetic LA. Moreover, we showed that treatment with safflower seed oil markedly enhanced proliferation of eNSCs, in comparison to LA or the control.

In accordance with our results, it has been previously shown that LA promotes the maintenance of embryonic NSPCs (Sakayori and Osumi, 2013). Moreover, another study reported the ability of arachidonic acid (ARA), a LA metabolite, to increase the number of neurospheres in neurogenic NSPCs (Sakayori et al., 2011). Our data and others suggested that LA is one of the potential fatty acids involved in the stimulation of proliferation. In order to confirm this, real time qPCR was used and showed an increase in mRNA expression levels of notch1, hes1, and Ki-67, suggesting an enhancement of stemness. In fact, Notch signaling is important in many aspects of CNS development (Louvi and Artavanis-Tsakonas, 2006), for instance, notch1 is essential for the maintenance of neural precursors, both *in vivo* and *in vitro* (Hitoshi et al., 2002). Indeed, the basic helix-loop-helix gene hes1, an essential effector of Notch signaling, regulates the maintenance of NSCs (Selkoe and Kopan, 2003). Interestingly, it has been reported that upon activation of Notch, NICD is released from the membrane and translocates to the nucleus to induce the expression of transcriptional repressor genes such as Hes1, which inhibits neuronal differentiation. Indeed, activation of Notch signaling leads to maintenance of the neural stem cell population (Bertrand et al., 2002; Imayoshi et al., 2010). In accordance, our data on the increase of Notch1 signaling pathway transcriptional expression was further investigated by examining NICD protein expression levels, which showed a similar trend in enhancing NSCs proliferation when low or high concentrations of safflower seed oil were used. These results confirm that safflower seed oil acts through Notch1 signaling pathway in order to enhance cell proliferation.

Safflower seed oil showed an increased potential in inducing proliferation, in comparison to LA, probably through the existence of OA and PA in this oil. Within the brain, PA has been reported as a main saturated fatty acid whereas OA as a main monounsaturated fatty acid (Bazinet and Laye, 2014). Substantial amount of data suggest both a protective (Prasad et al., 1998; Guan et al., 1999; Park et al., 2006) as well as deleterious effects (Wilson and Binder, 1997; Liu et al., 2004; Kim et al., 2005) of OA on some neurological disorders. For instance, two studies reported the beneficial role of OA on growth of axons and dendrites, facilitating neuronal migration (Tabernero et al., 2001; Medina and Tabernero, 2002). In addition, during myelination, the brain accumulates fatty acids associated with the myelin sheath, especially OA (Kuipers et al., 2012). Despite the beneficial results for LA and OA on neural cells, substantial studies have indicated that saturated fatty acids, including PA, caused toxicity in cells and various organs (Mu et al., 2001; Staiger et al., 2006; Evans et al., 2009), especially in numerous neurological disorders including ischemic, hypoxic, traumatic brain injury, and AD *in vivo* (Dhillon et al., 1999; Wu et al., 2003; Granholm et al., 2008). Recently, it has been demonstrated that high concentrations (50–500 μm) of PA suppressed the viability of NSCs and was cytotoxic to the cells (Wang et al., 2014). Moreover, *in vitro* studies demonstrated that PA induces neuronal apoptosis through intensifying cellular oxidative stress in rat cortical cells (Almaguel et al., 2009) whereas it was shown recently that PA causes NSCs apoptosis (Yuan et al., 2013).

In the current study, safflower seed oil showed a high potential in inducing proliferation at both low and high concentrations. Considering the beneficial role of OA in neural development and the deleterious role of PA, especially at high doses, we suggest that low concentrations of safflower seed oil are more potent than high concentrations, containing more OA and PA. Indeed, it seems that the beneficial effects of OA are reduced by the higher doses of PA through an unknown mechanism. We conclude that OA is the main factor responsible for enhancing LA ability in promoting the viability and proliferation of cells whereas PA is a dose-dependent suppressive factor, which attenuates the LA and OA effects.

After differentiation of cells, an increase in the number of GFAP, MBP, and β-III Tubulin positive cells was observed in all groups treated with safflower seed oil, in comparison to LA treated groups. However, this increase was only significant for neurons, but not for astrocytes and oligodendrocytes. In accordance, another study reported that conjugated LA (an isomer of LA), but not LA, promotes the neuronal differentiation of embryonic NSCs (Okui et al., 2011). Finally, treatment of neurogenic NSPCs by ARA did not show any change in differentiation conditions (Sakayori et al., 2011).

Neural stem cells are heterogeneous populations comprising bona fide stem cells and also progenitor cells of different neural cell lineages i.e., oligodendroglial, astrocytic, and neuronal progenitors (Azari and Reynolds, 2016). When cultured in a proliferative condition in the presence of growth factors, it appeared that safflower seed oil increased the proliferation and stemness property via Notch 1 signaling, as discussed earlier. Interestingly, when these cells were plated in a differentiating culture condition in the absence of growth factors, an overall increase of differentiation in all three neural lineages was noticed, of which only the neuronal cell differentiation was statistically significant, in comparison to controls. This could be due to an overall increase in cell viability in presence of safflower seed oil as shown in this study but the exact mechanisms need further investigations that were beyond the scope of the current study.

In conclusion, our results demonstrated the high beneficial potential of safflower seed oil on maintenance of the stemness of embryonic NSCs, in comparison to pure synthetic LA. To this end, safflower seed oil with natural abundance of OA and lower concentrations of PA and LA could be considered in diets and complementary medicines.

## Author contributions

MG, AG, and KZ designed the study and interpreted all data. KZ, HA, and MG wrote the paper. HH interpreted some data. FS and RJ prepared the real-time PCR samples. EN, GG, and HD participated in the collection of data.

### Conflict of interest statement

The authors declare that the research was conducted in the absence of any commercial or financial relationships that could be construed as a potential conflict of interest.

## Abbreviations

eNSCs: embryonic neural stem cells
LA: linoleic acid
OA: oleic acid
PA: palmitic acid
MBP: myelin basic protein
GFAP: glial fibrillary acidic protein
bHLH: basic helix-loop-helix
NICD: Notch intracellular domain.
